# A novel equation for the estimation of low-density lipoprotein cholesterol in the Saudi Arabian population: a derivation and validation study

**DOI:** 10.1038/s41598-024-55921-w

**Published:** 2024-03-05

**Authors:** Dena A. Nuwaylati, Zuhier A. Awan

**Affiliations:** 1 Department of Clinical Biochemistry, Faculty of Medicine, University of Jeddah, 21959 Jeddah, Saudi Arabia; 2 Department of Clinical Biochemistry, Faculty of Medicine, King Abdulaziz University, 21465 Jeddah, Saudi Arabia

**Keywords:** Lipids, Cardiology

## Abstract

Low-density lipoprotein cholesterol (LDL-C) is typically estimated by the Friedewald equation to guide atherosclerotic cardiovascular disease (ASCVD) management despite its flaws. Martin–Hopkins and Sampson-NIH equations were shown to outperform Friedewald’s in various populations. Our aim was to derive a novel equation for accurate LDL-C estimation in Saudi Arabians and to compare it to Friedewald, Martin–Hopkins and Sampson-NIH equations. This is a cross-sectional study on 2245 subjects who were allocated to 2 cohorts; a derivation (1) and a validation cohort (2). Cohort 1 was analyzed in a multiple regression model to derive an equation (equation^D^) for estimating LDL-C. The agreement between the measured (LDL-C^DM^) and calculated levels was tested by Bland–Altman analysis, and the biases by absolute error values. Validation of the derived equation was carried out across LDL-C and triglyceride (TG)-stratified groups. The mean LDL-C^DM^ was 3.10 ± 1.07 and 3.09 ± 1.06 mmol/L in cohorts 1 and 2, respectively. The derived equation is: LDL-C^D^ = 0.224 + (TC × 0.919) – (HDL-C × 0.904) – (TG × 0.236) – (age × 0.001) – 0.024. In cohort 2, the mean LDL-C (mmol/L) was estimated as 3.09 ± 1.06 by equation^D^, 2.85 ± 1.12 by Friedewald, 2.95 ± 1.09 by Martin–Hopkins, and 2.93 ± 1.11 by Sampson-NIH equations; statistically significant differences between direct and calculated LDL-C was observed with the later three equations (*P* < 0.001). Bland–Altman analysis showed the lowest bias (0.001 mmol/L) with equation^D^ as compared to 0.24, 0.15, and 0.17 mmol/L with Friedewald, Martin–Hopkins, and Sampson-NIH equations, respectively. The absolute errors in all guideline-stratified LDL-C categories was the lowest with equation^D^, which also showed the best classifier of LDL-C according to guidelines. Moreover, equation^D^ predicted LDL-C levels with the lowest error with TG levels up to 5.63 mmol/L. Equation^D^ topped the other equations in estimating LDL-C in Saudi Arabians as it could permit better estimation when LDL-C is < 2.4 mmol/L, in familial hyperlipidemia, and in hypertriglyceridemia, which improves cardiovascular outcomes in high-risk patients. We recommend further research to validate equation^D^ in a larger dataset and in other populations.

## Introduction

Cardiovascular diseases (CVD) remain the leading cause of mortality worldwide, claiming around 18 million lives annually^1,2^. The prevalence of atherosclerotic cardiovascular diseases (ASCVD), the most common CVD, is estimated to be approximately 6% among the Saudi Arabian population^2,3^, and a 2021 study reported that 50% of Saudi Arabians are hyperlipidemic^4^. The great health and economic costs associated with ASCVD are preventable by maintaining ideal blood lipids’ ranges^3,5, 6^.

Elevated low-density lipoprotein cholesterol (LDL-C) has been identified as a major risk factor for ASCVD, and attaining LDL-C treatment targets has successfully shown major cardiovascular (CV) risk and mortality reduction^7^. Therefore, LDL-C is considered the primary treatment target in all clinical practice guidelines for dyslipidemia management, which categorize CVD and adjust their treatment recommendations on the basis of LDL-C levels^8–10^. Moreover, LDL-C is a screening parameter for dyslipidemia among those with family history of genetic dyslipidemias, as well as chronic diseases with high CV risk^11^.

In light of this, it is not inexplicable to foresee the dire consequences of obtaining an inaccurate LDL-C level; an underestimated LDL-C can lead to suboptimal treatment and losing LDL-C target attainment, while contrarily, an overestimated LDL-C can induce preventable therapeutic adverse effects and burdens health care resources^5,6, 11, 12^. The gold standard reference measurement method for LDL-C is the direct beta quantification (βQ), which is based on ultracentrifugation for separating apolipoprotein-B (ApoB)-containing particles^13^, yet, it is a laborious, costly, slow process, and is not always convenient for routine laboratory use^14,15^.

Several years of endeavors to assure the accuracy of LDL-C estimation have resulted in the derivation of various equations as an alternative to the ßQ method, which was initiated by William Friedewald in 1972^16^. The Friedewald equation has been the most widely utilized in medical laboratories, yet it has some limitations. First and foremost, the equation does not account for interindividual variances in the triglyceride (TG):very-low-density lipoprotein cholesterol (VLDL-C) (TG:VLDL-C) ratio^17^; it is based on a presumption that this ratio is constantly 5:1, when in fact, it is highly variable through different TG and non-high-density lipoprotein cholesterol (non-HDL-C) levels^14,17^; the Lipid Research Clinics Prevalence Study reported a mean TG:VLDL-C ratio that ranges from 5.2 to 8.9 ^18^. Therefore, it is invalid at TG levels > 4.5 mmol/L^19^; particularly observed in diabetes mellitus (DM) and metabolic disorders^20^. Some studies have also questioned its accuracy with TG levels < 4.5 mmol/L and at optimal TG levels (< 0.80 mmol/L) ^20–22^. Additionally, it overestimates LDL-C levels in type III hyperlipoproteinemia with high VLDL^23^, and inaccurately estimates LDL-C in alcoholic liver cirrhosis and nephrotic syndrome^24^. Furthermore, it requires a fasting state, because the cholesterol-poor chylomicrons can overestimate VLDL-C in a non-fasting sample, which ultimately underestimates LDL-C^22^. Moreover, it can be inaccurate with low levels of LDL-C (< 2.4 mmol/L) ^12^, however, levels that low were not tested when the Friedewald equation was derived^16^. The Friedewald equation’s shortcomings have been tolerable for years, but nowadays, LDL-C reduction became easily sustainable with the evolution of lipid lowering regimens; levels < 2 mmol/L are seen with proprotein convertase subtilisin/kexin type 9 (PCSK9) inhibitors combined with statins^25^, and with the emerging small interfering RNA (siRNA) therapeutic agent (Inclisiran), LDL-C < 0.65 mmol/L was achieved^26^, which necessitates improving the accuracy of LDL-C estimating methods.

To overcome those setbacks, other equations have been formulated, some of which have also relied on a fixed factor for VLDL-C estimation, while others have not^17,27–32^. Of the most reliable ones are Martin–Hopkins and Sampson-NIH equations which were shown to improve the accuracy of LDL-C estimation as compared to Friedewald’s ^33^. However, the Friedewald equation is still being adopted in the majority of laboratories^33^.

Previous studies have shown that utilizing one equation to universally and equally predict LDL-C in all populations is not successful^12,34–36^. Accordingly, the equations derived over the years were population-specific. In our previous study^37^, we have validated the use of other equations in the Saudi Arabian population, and some were shown to be unsuccessful despite outperforming Friedewald’s in other populations. This could be explained by the genetic variations and possibly the variability in lifestyle factors that could both contribute to the divergence in lipid profiles among different populations^38,39^. Indeed, the prevalence of consanguineous marriages and familial hypercholesterolemia is among the highest in the world in the Saudi Arabian population^40,41^. In an attempt to improve LDL-C estimation, we endeavored to derive an equation that is specific to our population.

This study has two aims: first, to derive and validate a novel equation for estimating LDL-C from the standard lipid profile in the Saudi Arabian population, and second, to compare the directly measured LDL-C to those calculated by Friedewald, Martin–Hopkins and Sampson-NIH equations.

## Results

A total of 2245 subjects were included in the study. The majority of screened records were excluded; this was mainly due to the abundance of non-Saudi patients, the availability of lipid parameters tested on different dates, and the missing HDL-C levels in a large portion of subjects. Two-thirds comprised the derivation cohort (cohort 1; *n* = 1497), and one-third comprised the validation cohort (cohort 2; *n* = 748). The overall study population had a median age of 52 (21) years, and 61.7% were women. Both cohorts were homogenous in terms of age and lipid levels, while cohort 1 had more women (58.2%) than men (41.8%); *p* ≤ 0.001. The medians of the lipid parameters were: 4.92 (1.53) mmol/L for TC, 3.08 (1.36) mmol/L for measured LDL-C, 1.26 (0.48) mmol/L for HDL-C, and 1.44 (1.26) mmol/L for TG. Thirty seven percent of the samples were hyperlipidemic; of those, 36.1% had elevated LDL-C, 42.5% had hypertriglyceridemia, and 21.4% had mixed hyperlipidemia. The characteristics of the two cohorts are outlined in Table 1.

**Table 1:** Characteristics of the study population.

Males, *n* (%)	Total study population (n = 2245)		Cohort 1 (n = 1497)		Cohort 2 (n = 748)		P value < 0.001
	860 (38.3)		625 (41.8)		235 (31.4)		
	Median (IQR)	Min–Max	Median (IQR)	Min–Max	Median (IQR)	Min–Max	
Age (years)	52 (40–61)	18–95	53 (40–62)	18–91	51 (39–60)	18–95	0.060
TC (mmol/L)	4.92 (4.09–5.62)	1.22–20.26	4.92 (4.08–5.63)	1.22–20.26	4.92 (4.13–5.59)	1.91–13.49	0.984
LDL-C^DM^ (mmol/L)	3.08 (2.37–3.73)	0.13–11.04	3.09 (2.37–3.74)	0.13–11.04	3.03 (2.33–3.7)	0.94–10.31	0.686
HDL-C (mmol/L)	1.26 (1.04–1.52)	0.24–3.9	1.25 (1.03–1.52)	0.24–3.9	1.29 (1.07–1.53)	0.24–3.24	0.062
Non-HDL-C (mmol/L)	3.56 (2.78–4.28)	0.49–19.58	3.58 (2.76–4.3)	0.49–19.58	3.51 (2.81–4.26)	1.14–12.04	0.646
VLDL-C (mmol/L)	0.42 (0.22–0.70)	0.01–8.54	0.42 (0.23–0.69)	0.01–8.54	0.41 (0.22–0.71)	0.01–3.02	0.840
TG (mmol/L)	1.44 (0.94–2.2)	0.18–10.48	1.46 (0.95–2.24)	0.18–10.48	1.41 (0.94–2.08)	0.38–8.08	0.132
TG:VLDL-C	3.56 (2.51–5.58)	0.22–190	3.59 (2.54–5.55)	0.22–190	3.46 (2.39–5.67)	0.73–171	0.192
FBS (mmol/L)	5.6 (5.1–6.9)	2.70–33	5.7 (5.1–7.1)	3–33	5.6 (5.1–6.7)	2.7–32.4	0.058
HbA1C (%)	6 (5.5–7)	1.36–15.8	6 (5.5–7.3)	1.36–15.8	5.9 (5.5–6.7)	3.6–14	0.134

*TC* total cholesterol, *LDL-C*^*DM*^ directly measured low-density lipoprotein cholesterol, *HDL-C* high-density lipoprotein cholesterol, non-*HDL-C* non-high-density lipoprotein cholesterol, *VLDL-C* very-low-density lipoprotein cholesterol, *TG* triglycerides, *TG:VLDL-C* ratio of triglycerides to VLDL-C, *FBS* fasting blood sugar, *HbA1C* glycosylated hemoglobin.

Data presented as median with interquartile range (Q1–Q3) and minimum and maximum values (min–max) for continuous variables, and number (percentage) for the categorical variable (gender). Both cohorts were compared by Mann–Whitney U and chi-square tests.

**Figure 1:**
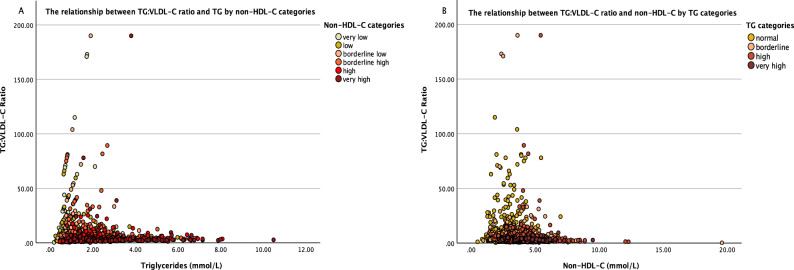
The distribution of TG:VLDL-C ratio: (**A**) in relation to triglycerides concentrations and color coded by non-HDL-C categories (darker dots imply higher non-HDL-C levels). (**B**) in relation to non-HDL-C concentrations and color coded by triglycerides categories (darker dots imply higher TG levels). TG and non-HDL-C categories are based on the ACC/AHA guideline classification.

TG:VLDL-C ratio showed very high inter-individual variations in our dataset, with a minimum value of 0.22 and a maximum of 190. Figure 1A shows the distribution of TG:VLDL-C ratios in relation to TG concentrations and across non-HDL-C categories. At low TG levels (approximately < 2 mmol/L), TG:VLDL-C ratios showed some elevation with increasing TG, however, this observation was lost as TG increased, and the overall ratios were independent of non-HDL-C levels. This relationship was further explored after log-transformation of TG:VLDL-C ratio and TG (Fig. S1 in the supplementary file); the plot failed to explain a clear pattern for their relationship, which was also independent of non-HDL-C concentrations. Figure 1B confirms the independence of the TG:VLDL-C ratio on non-HDL-C. At high TG levels (marked by the darker dots), the ratio is no longer consistently increasing with TG concentrations.

### Derivation of the equation

Data from cohort 1 was used in a multiple regression model to derive an equation for LDL-C calculation from the standard lipid profile, age, and gender that is independent of TG:VLDL-C ratio. There was a strong association between the measured LDL-C values and those predicted by the model (multiple correlation coefficient R = 0.97), and 94.5% of the independent variables collectively explained LDL-C concentrations (adjusted R^2^ = 0.945). The model significantly predicted LDL-C levels (*p* ≤ 0.001). The contribution of gender to the overall prediction was not statistically significant, and the prediction by the equation did not significantly alter by distinguishing corrective factors for each gender. Hence, due to the slightly more accurate prediction for LDL-C with using the 0.024 as a corrective factor for the female gender, and to simplify our equation’s applicability, we have used this factor all over the dataset.

The derived equation is:

$${\text{LDL}} - {\text{C}}^{{\text{D}}} \left( {{\text{mmol}}/{\text{L}}} \right) \, = \, 0.{224 } + \, \left( {{\text{TC }} \times \, 0.{919}} \right) \, {-} \, \left( {{\text{HDL}} - {\text{C}} \times \, 0.{9}0{4}} \right) \, {-} \, \left( {{\text{TG }} \times \, 0.{236}} \right) \, {-} \, \left( {{\text{age }} \times \, 0.00{1}} \right) \, {-} \, 0.0{24}{\text{.}}$$

### Validation of the derived equation

Table 2 compares the means of LDL-C^DM^ to those estimated by the equations. There was no statistically significant difference between LDL-C^DM^ and LDL-C^D^ in both cohorts (*p*≥ 0.05), while LDL-C estimated by the remaining equations showed a significant difference when compared to the direct method (*p* ≤ 0.001).

**Table 2:** LDL-C levels estimated by the equations in each cohort.

LDL-C (mmol/L)	Total population (*n* = 2445)	*P* value	Cohort 1 (*n* = 1497)	*P* value	Cohort 2 (*n* = 748)	*P* value
LDL-C^DM^	3.10 ± 1.07	–	3.10 ± 1.07	–	3.09 ± 1.06	–
LDL-C^D^	3.09 ± 1.08	0.146	3.09 ± 1.09	0.105	3.09 ± 1.06	0.912
LDL-C^F^	2.83 ± 1.14	< 0.001	2.83 ± 1.14	< 0.001	2.85 ± 1.12	< 0.001
LDL-C^M^	2.94 ± 1.16	< 0.001	2.94 ± 1.12	< 0.001	2.95 ± 1.09	< 0.001
LDL-C^S^	2.92 ± 1.12	< 0.001	2.91 ± 1.13	< 0.001	2.93 ± 1.11	< 0.001

Values presented as mean ± SD.* P* values are of paired t-test between direct LDL-C and LDL-C estimated by each equation. LDL-C^DM^, directly measured low-density lipoprotein cholesterol, LDL-C^D^, novel equation-derived LDL-C, LDL-C^F^, LDL-C calculated by Friedewald equation, LDL-C^M^, LDL-C calculated by Martin–Hopkins equation, LDL-C^S^, LDL-C calculated by Sampson-NIH equation.

**Figure 2:**
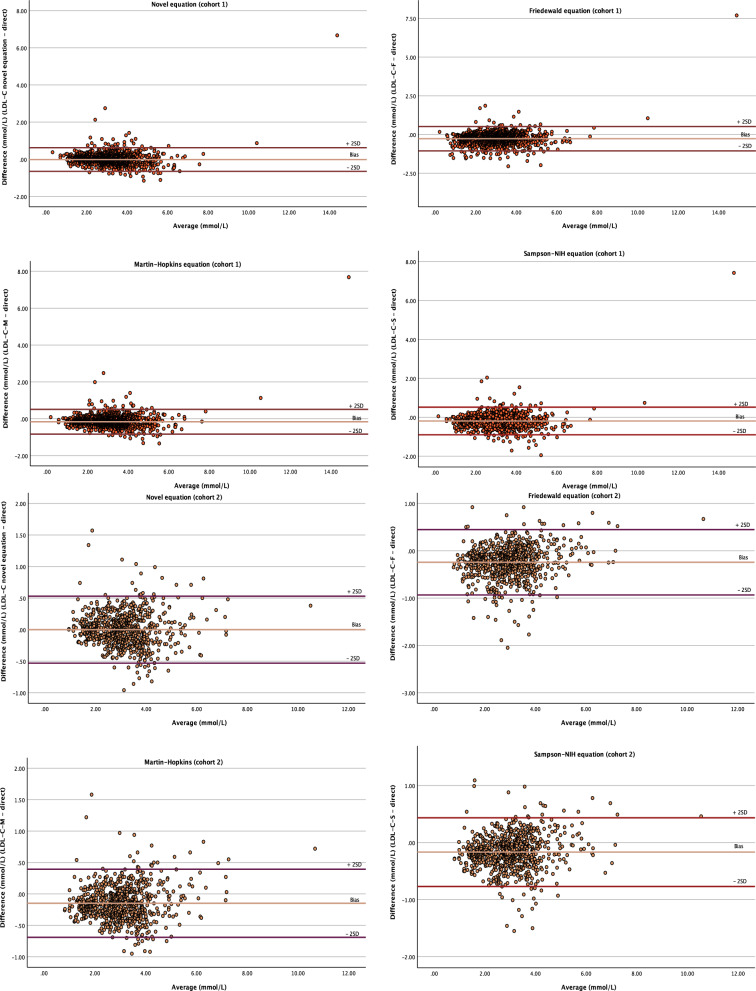
Bland–Altman plots of agreement between the directly measured and calculated LDL-C by the equations in both cohorts. The lines demonstrate the mean bias and 95% limits of agreement (± 2SD). *LDL-C* low-density lipoprotein cholesterol, *SD* standard deviation.

Bland–Altman analysis tested the agreement between the direct and calculated LDL-C by the equations. Figures 2 and S2 illustrate Bland–Altman plots; the middle line (light colored) shows the mean bias of each equation in both cohorts. The Friedewald, Martin–Hopkins, and Sampson-NIH equations underestimated LDL-C by approximately 0.24, 0.15, and 0.17 mmol/L, respectively, while equation^D^ showed a lower bias that was close to zero; approximately 0.001 mmol/L (Table 3).

**Table 3:** Bland–Altman analysis: mean bias with the limits of agreement (LOA).

	Whole population			Derivation cohort			Validation cohort		
	Bias	Lower LOA	Upper LOA	Bias	Lower LOA	Upper LOA	Bias	Lower LOA	Upper LOA
Equation^*D*^	–0.01	–0.61	0.59	–0.01	–0.65	0.62	–0.001	–0.53	0.53
Friedewald equation	–0.26	–1.02	0.49	–0.27	–1.06	0.52	–0.24	–0.93	0.45
Martin–Hopkins equation	–0.16	–0.79	0.47	–0.16	–0.83	0.51	–0.15	–0.69	0.39
Sampson-NIH equation	–0.18	–0.86	0.49	–0.19	–0.90	0.52	–0.17	–0.77	0.43

Bias and LOA are presented as mmol/L.

*LOA* limits of agreement (corresponds to ± 2 standard deviations from the mean).

The performance of the equations was further tested by calculating the magnitude of error for all LDL-C values which was assessed in the predefined LDL-C stratified groups. The percentage of samples by the magnitude of error between direct and calculated LDL-C stratified by LDL-C categories is outlined in Table 4. Upon focus on the validation cohort, and across all LDL-C categories, equation^D^ estimated LDL-C with the lowest bias as compared to Friedewald, Martin–Hopkins, and Sampson-NIH equations, while Friedewald showed the lowest accuracy with the highest magnitude of error among all of them.

**Table 4:** The percentage of samples by the magnitude of error between direct and calculated LDL-C by guideline-classified LDL-C categories in both cohorts.

Absolute error (mmol/L)	Percentage of samples in the whole population				Percentage of samples in cohort 1				Percentage of samples in cohort 2			
	Equation^D^	Friedewald	Martin–Hopkins	Sampson-NIH	Equation^D^	Friedewald	Martin–Hopkins	Sampson-NIH	Equation^D^	Friedewald	Martin–Hopkins	Sampson-NIH
LDL-C < 1.92 mmol/L												
	n = 350				n = 239				n = 111			
< 0.13	58.9	27.4	34.6	28.6	57.3	25.5	36.0	29.3	62.2	31.5	31.5	27.0
0.13–0.24	26.3	29.7	32.6	34.9	27.6	31.0	32.6	35.1	23.4	27.0	32.4	34.2
0.25–0.50	12.6	27.7	30.0	30.6	13.0	28.0	28.5	29.7	11.7	27.0	33.3	32.4
0.51–0.76	0.6	10.9	0.9	4.6	0.4	10.9	0.8	4.6	0.9	10.8	0.9	4.5
> 0.77	1.7	4.3	2.0	1.4	1.7	4.6	2.1	1.3	1.8	3.6	1.8	1.8

LDL-C 1.93–2.57 mmol/L												
	n = 331				n = 218				n = 113			
< 0.13	53.2	26.9	29.3	29.6	54.6	27.5	29.8	27.5	50.4	25.7	28.3	33.6
0.13–0.24	25.4	24.2	33.8	28.4	24.8	21.6	32.1	28.4	26.5	29.2	37.2	28.3
0.25–0.50	18.7	33.5	33.8	35.6	17.4	34.4	34.9	37.2	21.2	31.9	31.9	32.7
0.51–0.76	1.8	8.5	2.1	3.9	2.3	8.7	2.3	3.7	0.9	8.0	1.8	4.4
> 0.77	0.9	6.9	0.9	2.4	0.9	7.8	0.9	3.2	0.9	5.3	0.9	0.9

LDL-C 2.58–3.22 mmol/L												
	n = 594				n = 390				n = 204			
< 0.13	42.3	23.9	31.0	29.5	42.6	23.8	30.0	27.9	41.7	24.0	32.8	32.4
0.13–0.24	32.2	19.4	24.1	23.1	32.8	18.7	24.1	24.4	30.9	20.6	24.0	20.6
0.25–0.50	22.1	36.2	39.6	36.4	20.0	38.2	41.3	37.9	26.0	32.4	36.3	33.3
0.51–0.76	2.7	12.3	5.1	9.3	3.6	12.6	4.4	7.9	1.0	11.8	6.4	11.8
> 0.77	0.8	8.2	0.3	1.9	1.0	6.7	0.3	1.8	0.5	11.3	0.5	2.0

LDL-C 3.23–3.86 mmol/L												
	n = 493				n = 328				n = 165			
< 0.13	37.5	25.2	25.8	29.0	39.6	23.2	25.0	28.4	33.3	29.1	27.3	30.3
0.13–0.24	30.2	19.9	28.8	23.7	29.9	19.2	29.3	21.3	30.9	21.2	27.9	28.5
0.25–0.50	27.2	34.5	37.5	36.7	25.9	35.1	36.9	39.0	29.7	33.3	38.8	32.1
0.51–0.76	3.2	15.6	6.5	8.1	2.7	16.8	7.3	8.2	4.2	13.3	4.8	7.9
> 0.77	1.8	4.9	1.4	2.4	1.8	5.8	1.5	3.0	1.8	3.0	1.2	1.2

LDL-C 3.87–4.5 mmol/L												
	n = 306				n = 208				n = 98			
< 0.13	29.1	17.3	21.2	20.3	30.8	18.3	24.0	20.2	25.5	15.3	15.3	20.4
0.13–0.24	27.5	19.3	21.9	22.2	27.9	19.7	23.1	25.5	26.5	18.4	19.4	15.3
0.25–0.50	34.0	36.6	40.2	38.2	33.7	38.5	39.9	38.5	34.7	32.7	40.8	37.8
0.51–0.76	7.2	17.0	14.4	12.7	5.8	15.4	11.5	10.1	10.2	20.4	20.4	18.4
> 0.77	2.3	9.8	2.3	6.5	1.9	8.2	1.4	5.8	3.1	13.3	4.1	8.2

LDL-C ≥ 4.5 mmol/L												
	n = 171				n = 114				n = 57			
< 0.13	22.2	15.8	20.5	18.1	16.7	12.3	16.7	14.9	33.3	22.8	28.1	24.6
0.13–0.24	25.1	17.0	15.2	17.0	26.3	14.9	14.0	14.0	22.8	21.1	17.5	22.8
0.25–0.50	33.3	33.3	38.6	33.9	35.1	36.0	41.2	36.0	29.8	28.1	33.3	29.8
0.51–0.76	14.6	17.0	17.0	17.5	16.7	16.7	16.7	19.3	10.5	17.5	17.5	14.0
> 0.77	4.7	17.0	8.8	13.5	5.3	20.2	11.4	15.8	3.5	10.5	3.5	8.8

The numbers present the percentage of subjects in each category. LDL-C categories are classified based on the ACC/AHA guideline.

At LDL-C levels of < 1.92 mmol/L, equation^D^ estimated LDL-C with the lowest error (≤ 0.24 mmol/L) in 85% of subjects as compared to 58% with Friedewald, 64% with Martin-Hopkins, and 61% with Sampson-NIH equations. Additionally, an error of ≥ 0.77 mmol/L in this group was seen with only 1.8% of LDL-C estimated by equation^D^, Martin–Hopkins and Sampson-NIH equations, which were lower than Friedewald’s (3.6%). In the mid-ranges of LDL-C levels (1.93–2.57, 2.58–3.22, and 3.23–3.86 mmol/L), equation^D^ also predicted LDL-C with the lowest errors as compared to the other equations; approximately 70% of LDL-C were predicted with an error between 0 and 0.24 mmol/L, as compared to approximately 50% with Friedewald and 59% with both Martin–Hopkins and Sampson-NIH equations. Larger errors (> 0.24 mmol/L) among these ranges were also observed in a smaller percentage of LDL-C calculated by equation^D^ as compared to the other equations.

At higher LDL-C levels (≥ 3.87 mmol/L), an error of ≥ 0.77 mmol/L was the lowest with equation^D^ (3%), followed by Martin–Hopkins (4%), Sampson-NIH (8%), then with Friedewald (12%) equation which was associated with the highest error. Furthermore, the highest percentage of subjects with the lowest error (≤ 0.24 mmol/L) in these groups was also observed with equation^D^ (53%), followed by Sampson-NIH (40%), Martin–Hopkins (39%), and finally Friedewald (37%) equations.

**Figure 3:**
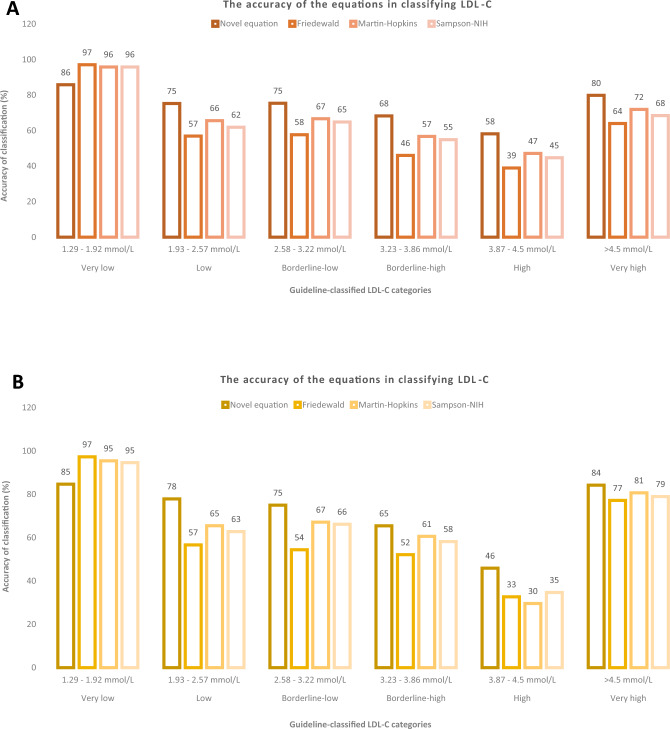
The accuracy of the equations in classifying LDL-C according to the guideline categories in: (**A**) derivation cohort and (**B**) validation cohort.

Moreover, the accuracy of the equations, as determined by their ability to correctly classify LDL-C according to guideline-stratified categories, was calculated in percentage. In both cohorts, and in all categories, except for LDL-C < 1.92 mmol/L, equation^D^ showed the best classification for LDL-C among the equations, followed by Martin–Hopkins, Sampson-NIH, and finally Friedewald equations, which showed the least appropriate classifier for all LDL-C categories (Fig. 3 and S3).

**Figure 4:**
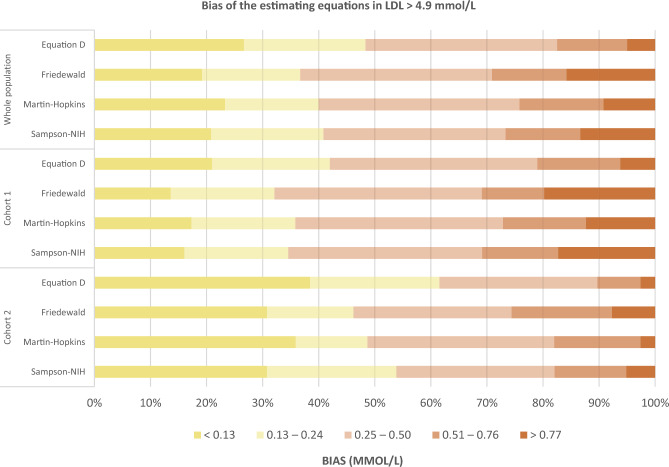
The bias of the equations in estimating LDL-C ≥ 4.9 mmol/L (yellow colors denote the lowest errors of < 0.24 mmol/L).

The magnitude of error by the equations was sub-analyzed in a group of samples with LDL-C levels ≥ 4.9 mmol/L (Fig. 4 and Table S2). Similarly, the lowest bias in LDL-C estimation was observed with equation^D^; 61% of subjects were estimated with an error of ≤ 0.24 mmol/L, as compared to 53.8% with Sampson-NIH, 48.7% with Martin–Hopkins, and 46.2% with Friedewald equations. Higher errors of ≥ 0.51 mmol/L were seen with only 10.3% of subjects with equation^D^, as compared to 18% with both Martin–Hopkins and Sampson-NIH equations and 25.6% with Friedewald equation. Moreover, the misclassification of LDL-C at this level was the lowest with equation^D^ as compared to the other three equations (Fig. S4).

**Figure 5:**
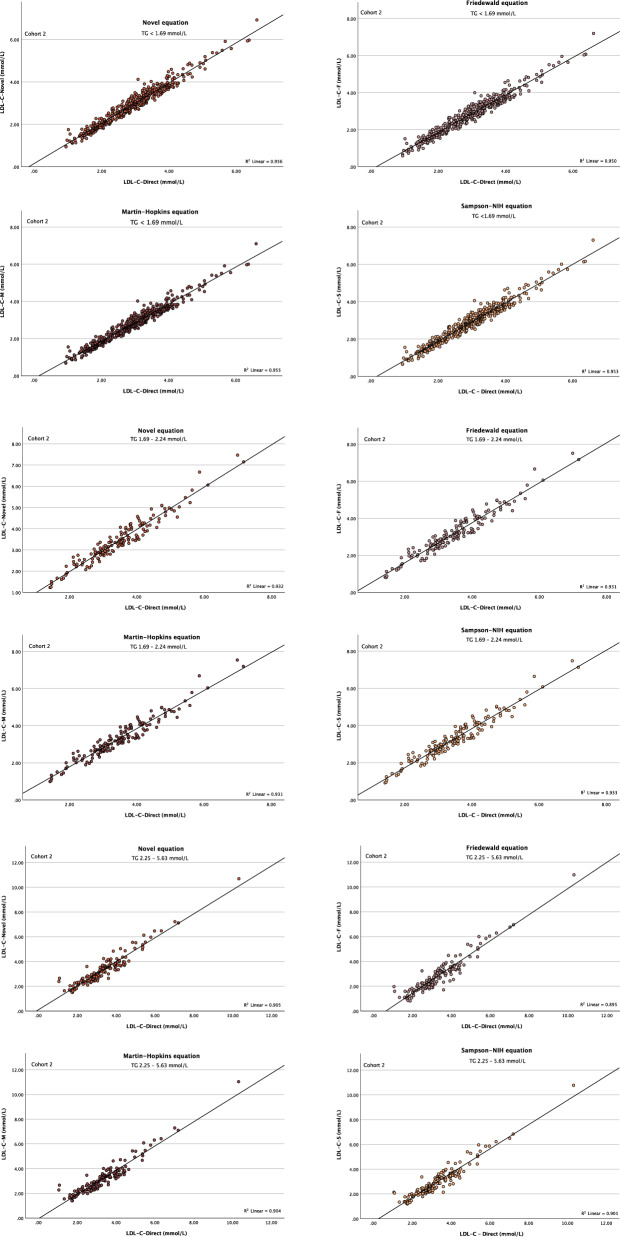
The correlation between direct and calculated LDL-C across TG categories in the validation cohort.

Finally, the performance of the equations was assessed in different TG categories. Across all TG levels up to 5.63 mmol/L, LDL-C^DM^ strongly correlated with the estimated LDL-C by the four equations, with a slightly higher correlation with equation^D^ in most groups (Figs. 5 and S5-S10), which was also associated with the lowest mean bias (Tables S3 and S4). With TG levels ≥ 5.64 mmol/L, the lowest mean bias between the direct and calculated LDL-C was observed with equation^D^ and Martin–Hopkins as compared to other two equations, which was slightly lower with Martin–Hopkins equation (0.01 mmol/L) in cohort 1, and with equation^D^ (0.06 mmol/L) in cohort 2 (Table S4).

The percentage of samples by the magnitude of error between direct and calculated LDL-C stratified by TG categories is outlined in Table 5. Equation^D^ predicted LDL-C with the lowest magnitude of error in all TG levels among the four equations. At high TG levels (2.25–5.63 mol/L), equation^D^ estimated 53% of subjects with a low error (≤ 0.24 mmol/L), as compared to 48% with Martin–Hopkins, 33% with Sampson-NIH, and 21% with Friedewald equations. Moreover, in this category, the highest error of ≥ 0.77 mmol/L was observed with Friedewald followed by Sampson-NIH equations in 28% and 11% of subjects, respectively. While only 5% of subjects were predicted with high errors with both equation^D^ and Martin–Hopkins equation. At TG levels ≥ 5.64 mmol/L, Friedewald equation failed to predict any LDL-C levels with errors ≤ 0.24 mmol/L, and 77% of subjects were predicted with an error ≥ 0.77 mmol/L. The lowest error (≤ 0.24 mmol/L) was observed with equation^D^ in 22% of subjects as compared to 11% with Martin–Hopkins and Sampson-NIH equations. While all of the equations were associated with higher errors at this level, equation^D^ and Martin–Hopkins equation showed better predictive abilities than Sampson-NIH equation, which predicted 44% of subjects with an error of ≥ 0.77 mmol/L.

**Table 5:** The percentage of samples by the magnitude of error between direct and calculated LDL-C by guideline-classified TG categories in both cohorts.

	Percentage of samples in the whole population				Percentage of samples in cohort 1				Percentage of samples in cohort 2			
Absolute error (mmol/L)	Equation^D^	Friedewald	Martin–Hopkins	Sampson-NIH	Equation^D^	Friedewald	Martin–Hopkins	Sampson-NIH	Equation^D^	Friedewald	Martin–Hopkins	Sampson-NIH
TG < 1.69 mmol/L												
	n = 1290				n = 846				n = 444			
< 0.13	49.3	32.6	28.9	34.2	49.3	32.6	28.8	34.2	49.5	32.7	29.3	34.2
0.13–0.24	29.9	28.8	30.7	31.2	30.5	28.7	30.7	31.2	28.8	29.1	30.9	31.1
0.25–0.50	19.1	34.6	37.9	32.6	18.4	34.9	38.1	32.5	20.5	34.2	37.8	32.7
0.51–0.76	1.2	3.5	2	1.8	1.4	3.4	2.1	1.8	0.9	3.8	1.8	1.8
> 0.77	0.3	0.3	0.2	0.3	0.4	0.4	0.2	0.4	0.2	0.2	0.2	0.2

TG 1.69–2.24 mmol/L												
	n = 426				n = 277				n = 149			
< 0.13	32.9	15.0	25.8	21.8	33.6	12.6	25.3	19.5	31.5	19.5	26.8	26.2
0.13–0.24	30.3	16.4	21.1	20.9	31.0	16.2	21.3	22.4	28.9	16.8	20.8	18.1
0.25–0.50	30.5	40.1	41.5	43.0	28.5	43.7	43.0	44.4	34.2	33.6	38.9	40.3
0.51–0.76	5.2	23.7	10.3	13.1	5.4	22.7	9.0	12.3	4.7	25.5	12.8	14.8
> 0.77	1.2	4.7	1.2	1.2	1.4	4.7	1.4	1.4	0.7	4.7	0.7	0.7

TG 2.25–5.63 mmol/L												
	n = 502				n = 356				n = 146			
< 0.13	32.5	9.2	28.3	14.3	34.3	8.7	28.9	12.9	28.1	10.3	26.7	17.8
0.13–0.24	24.7	8.4	21.5	14.5	24.4	7.3	21.6	14.3	25.3	11.0	21.2	15.1
0.25–0.50	29.5	27.9	30.3	38.0	28.9	30.3	30.9	41.9	30.8	21.9	28.8	28.8
0.51–0.76	9.2	29.1	14.3	21.1	8.7	29.5	12.9	18.5	10.3	28.1	17.8	27.4
> 0.77	4.2	25.5	5.6	12.0	3.7	24.2	5.6	12.4	5.5	28.8	5.5	11.0

TG > 5.64 mmol/L												
	n = 27				n = 18				n = 9			
< 0.13	18.5	0	11.1	11.1	16.7	0	11.1	11.1	22.2	0	11.1	11.1
0.13–0.24	14.8	3.7	33.3	11.1	16.7	5.6	33.3	16.7	11.1	0	33.3	0.0
0.25–0.50	25.9	14.8	25.9	11.1	22.2	16.7	27.8	11.1	33.3	11.1	22.2	11.1
0.51–0.76	11.1	14.8	11.1	29.6	11.1	16.7	11.1	27.8	11.1	11.1	11.1	33.3
> 0.77	29.6	66.7	18.5	37.0	33.3	61.1	16.7	33.3	22.2	77.8	22.2	44.4

The numbers present the percentage of subjects in each category. TG categories are classified based on the ACC/AHA guideline.

## Discussion

Early detection of dyslipidemia is the key for ASCVD prevention that also mitigates its associated health and economic costs. Accurate LDL-C estimation is fundamental to classify CVD and initiate the suitable therapeutic intervention, nevertheless, it remains a very common challenge in the medical laboratory^12^. Several efforts have been made to overcome the barriers of safely implementing the Friedewald equation in different high CV risk conditions, such as the metabolically compromised states with hypertriglyceridemia (TG > 4.5 mmol/L), in familial hyperlipidemia (LDL ≥ 4.9 mmol/L) with a high risk of premature myocardial infarctions as well as in very low LDL-C levels^12^.

Despite the various trials to derive an accurate LDL-C estimating method, Friedewald and others have formulated equations that relied on the concept that TG:VLDL-C ratio is constant; for example, a factor of 5, 6.85, and 6 have been suggested^16,28, 32^. This perception is the source of error in these equations. VLDL-C is highly variable; TG content within VLDL particles ranges from 50 to 70% while 10–25% is comprised of cholesteryl esters^42^, hence, assuming a fixed ratio is erroneous, and would never attribute to TG:VLDL-C ratio’s variability. Furthermore, the fluctuation of VLDL particle sizes also influences its lipid content and their relation to one another, which also limits relying on this ratio^43^. Moreover, VLDL-C can be inaccurately quantified in hypertriglyceridemic samples because the TG-rich lipoproteins (TRLs) are prone to sticking to centrifugation tubes, hence their possible false low levels, and LDL-C estimating equations based on a fixed or an adjustable factor that falls within a narrow range for TG:VLDL-C derived from these samples only maximizes the error^43^.

Based on the assumption that VLDL-C can be almost accurately calculated in fasting states, we calculated VLDL-C and found that using a “constant” factor to substitute “5” (or 2.2 when mmol/L is used) in Friedewald equation to reflect TG:VLDL-C in every individual is not possible, even with ideal TG levels. This factor was shown to be highly inconsistent between individuals; the mean TG:VLDL-C ratio in our study population was 6.03, and it ranged from 0.22 to 190. Figure 1 confirms the high variability of the ratio in relation to the status of TG and non-HDL-C concentrations; the two parameters that mostly reflect the core lipids that can alter this ratio^17^. The independence of TG:VLDL-C ratio on non-HDL-C was also observed in Martin’s 180-cell table of the medians of TG:VLDL-C ratios across different TG and non-HDL-C concentrations, and was also in-line with Sampson’s finding^17,43^. Ideally, higher VLDL-TG increases with mild TG elevation due to TG incorporation and storage within VLDL-C particles, which in turn causes its cholesterol content to appear lower, hence elevates the ratio. However, with further TG elevation, the loss of this observation might be explained by other factors affecting the TG:VLDL-C. Accordingly, we sought to derive a novel method for accurate LDL-C estimation that is independent of TG:VLDL-C. Direct LDL-C was quantified in our laboratory using a standard homogenous enzymatic method, which has been previously shown to be highly correlated with the ultracentrifugation (βQ) method^45^ and has been adopted as a practical gold standard for direct LDL-C estimation that is widely employed in research as the reference for LDL-C estimating equations^12,46–49^.

Using the standard lipid profile, age and gender, we were able to derive an equation that surpassed Friedewald, Martin–Hopkins, and Sampson-NIH equations in estimating LDL-C in our population. Overall, equation^D^ showed the highest agreement with LDL-C^DM^ among the four equations in both cohorts. It was associated with the lowest bias that was close to zero (0.001 mmol/L) as compared to Friedewald (0.24 mmol/L), Martin–Hopkins (0.15 mmol/L), and Sampson-NIH (0.17 mmol/L) equations. The largest percentage of subjects with the highest magnitude of error was observed with the Friedewald equation, followed by Martin–Hopkins and Sampson-NIH equations. On the other hand, a magnitude of error of > 0.24 mmol/L was the least observed with equation^D^ as compared to the other three methods. According to the guideline LDL-C classification, the very-low LDL-C category (< 1.92 mmol/L) was estimated with an error of > 0.24 mmol/L in only 14% of subjects with equation^D^, as compared to 41% with Friedewald, 36% with Martin–Hopkins, and 39% with Sampson-NIH equations. Low and borderline LDL-C-ranges (1.93–3.86 mmol/L) were 22% more accurately predicted by equation^D^ than Friedewald, and 12% and 14% better than Martin–Hopkins and Sampson-NIH equations, respectively. At levels ≥ 3.87 mmol/L, LDL-C estimated by equation^D^ was also more accurate than the other equations by approximately 15%. Despite its association with the lowest errors among the equations, equation^D^ still predicted a few samples with an error of > 0.24 mmol/L. This was observed in higher LDL-C categories as the number of samples dropped; which suggests that a larger sample might be required for the equation to be validated.

Furthermore, our equation’s accuracy has shown a lower misclassification of LDL-C according to the guideline categories. In all LDL-C classes, except for very-low, the misclassification was the lowest with equation^D^; around 70% of samples were correctly classified by equation^D^ as compared to approximately 60–65% by the other equations. The slightly lower percentage observed with equation^D^, Martin–Hopkins, and Sampson-NIH equations as compared to Friedewald in the very-low class of LDL-C however, does not imply their lower accuracy; as the Friedewald equation better classified LDL-C in this class of LDL-C only, it was in fact associated with a higher amount of error. Thirty-eight subjects in this category were misclassified by our equation but not by Friedewald. A possible explanation is the smaller sample size in this category that actual percentages were not detected. Furthermore, estimating equations are prone to misclassify LDL-C at certain TG levels even with very low LDL-C^32,50–52^. This was observed in this category, but could not be ascertained because our data did not yield a sufficient sample with very high TG levels to be accounted for by the derived equation. Moreover, a small percentage of correctly classified samples by all the four equations was observed in the high LDL-C category, however, a clear explanation cannot be elucidated.

Our sub-analysis on the clinically established LDL-C cutoff for the diagnosis of familial hyperlipidemia (FH) (≥ 4.9 mmol/L) ^53^ showed that equation^D^ predicted LDL-C with the lowest bias and it was associated with the least misclassification for this level among the other equations. This illustrates the impact of our equation in promoting primary cardiovascular prevention among those with genetic hyperlipidemia at high CV risk, which in turn aids in the reduction of their associated health and economical burden.

Linear regression showed a high correlation between LDL-C^DM^ and those calculated by the equations in different TG concentrations up to 5.63 mmol/L. However, since these correlations do not guarantee better LDL-C prediction, we have looked at their absolute errors within TG-stratified groups. In TG levels up to 5.63 mmol/L, equation^D^ was associated with the lowest error, followed by Martin–Hopkins, Sampson-NIH, then Friedewald equations. However, due to the overall very small number of subjects with TG > 5.63 mmol/L, a clear relationship between direct and estimated LDL-C could not be observed, and the performance of these equations at this TG level could not be validated.

The inaccuracy of the Friedewald equation and the superiority of others over it have been previously documented in other populations^12,29, 54, 55^, which is in line with our findings. In our previous study^37^, we have tested the performance of other equations in addition to Friedewald; Cordova, Hata, Puavilai, Chen, Ahmadi, Hattori, and Vujovic equations, and none have shown an error as low as equation^D^.

The impact of our equation on clinical outcomes and patients’ care is paramount, because the accuracy of LDL-C estimation is directly linked to the correct adjustment of ASCVD treatment, and therefore CV risk reduction. Large LDL-C under/overestimations can have life-threatening effects from treatment delays or unnecessary therapies, and our equation can overcome this matter among Saudi Arabians, even among FH patients.

Despite the worth of obtaining a population-specific LDL-C estimating equation for our population, its performance on non-Saudi Arabians cannot be currently ascertained. Alternatively, we recommend the current implementation of the Martin–Hopkins equation for non-Saudis in our laboratories due to its previous validation in different populations and its superiority to Friedewald’s. Furthermore, we recommend validating equation^D^ in a larger dataset, in non-Saudis residing in our region, and in other populations to test its performance.

To the best of our knowledge, this is the first study to derive a population-specific equation for LDL-C estimation in Saudi Arabians. Moreover, our sample size is nearly five times larger than Friedewald’s and various other similar studies^55^. This study has some limitations. The LDL-C was directly measured in our laboratory using the homogenous enzymatic assay rather than the gold standard βQ method. Some homogenous assays could be associated with poor analytical performance in certain diseases. Moreover, they may not exhibit complete LDL-C specificity in the presence of abnormal lipoproteins, and its accuracy could sometimes be influenced by elevated TGs^56,57^. As shown by some studied, the homogenous assays might also be sometimes discordant with the ßQ method at low LDL-C levels^58^. Calculated VLDL-C levels can also reflect intermediate-density lipoprotein cholesterol (IDL-C) and lipoprotein (a) (Lp(a)), which constitutes a negligible amount of non-HDL-C, except in the very rare cases of elevated Lp(a) and type III dyslipidemia, which were not captured from our records, however, due to their rarity, these cases are not expected to alter our VLDL-C values and change the observed TG:VLDL-C ratio, and since our equation is independent of VLDL-C, it certainly does not impact its accuracy. Moreover, our study could not include a sufficient sample with TG levels > 5.63 mmol/L, hence, our equation was not tested in severe hypertriglyceridemia. Finally, a larger sample is required to validate its performance across all LDL-C and TG levels before its employment in clinical practice.

In conclusion, our novel equation outperformed other equations in estimating LDL-C across its levels, and in TG as high as 5.63 mmol/L, which can promote CV risk prevention and lessen the healthcare costs for those with familial hyperlipidemia. We recommend validating equation^D^ on a bigger sample size, in other populations, and in severe hypertriglyceridemia before its implementation in clinical laboratories.

## Methods

### Study design and subjects’ selection

This is a cross-sectional, data-based, derivation and validation study that evaluated the accuracy of the Friedewald, Martin–Hopkins, and Sampson-NIH equations and derived a novel equation for LDL-C estimation in Saudi Arabians following up in King Abdulaziz University Hospital (KAUH), Jeddah, Saudi Arabia. Medical records were screened for LDL-C tested between 2009 and 2022. The inclusion criteria were: Saudi Arabian subjects of both genders, aged ≥ 18 years old, tested for complete fasting lipid profile. The origin of ethnicity was indicated in the demographics section of the medical record of each subject. The exclusion criteria were: subjects with non-fasting samples, missing lipid parameters, and subjects with lipid parameters tested on different occasions. Blood samples were not collected for lipid testing for non-fasting subjects; this was indicated on the lipid panel results for non-fasting samples in our laboratory. To augment the general applicability of our results, we included both normolipemic and hyperlipidemic subjects, both genders, and no TG level restrictions were applied. The total study population was randomly allocated to either a derivation or a validation data set; cohorts 1 and 2, respectively. Both cohorts comprised a wide range of LDL-C and TG levels. This study was performed in accordance with the Declaration of Helsinki principles. The institutional review board of King AbdulAziz University approved our study (Reference No. 314/22). An Informed consent was waived by the Research Ethics Committee (REC) of the Unit of Biomedical Ethics of King AbdulAziz University. This was mainly due to the nature of our study as we have used electronic-based data that posed no risks to the study subjects, in addition to the impracticality of obtaining consents for samples taken during the preceding years.

### Data collection and biochemical measurements

Data extraction was performed electronically. The four major lipid parameters were obtained in mmol/L: total cholesterol (TC), TG, LDL-C, and HDL-C. Non-HDL-C levels were calculated by: [TC − HDL-C]. LDL-C levels were also calculated by the Friedewald equation as: LDL-C^F^ (mmol/L) = [TC − HDL − TG/2.2] ^16^, by Martin–Hopkins equation (LDL-C^M^) as: [TC − HDL − (TG/adjustable factor)] through an excel-based automated calculator (available online through Johns Hopkins School of Medicine: https://ldlcalculator.com/) ^17^, and by Sampson-NIH equation (LDL-C^S^) as: (TC/0.948) − (HDL-C/0.971) − [(TG/8.56) + ((TG × non-HDL-C)/2140)–(TG^2^/16,100)]–9.44 ^43,44^. For both Martin–Hopkins and Sampson-NIH equations, LDL-C was calculated in mg/dL, which was then converted to mmol/L. Since the samples were obtained from fasting subjects, where chylomicrons and their remnants are nearly non-existent, VLDL-C levels were calculated as: [TC − HDL-C − LDL-C] ^59^. Fasting blood sugar (FBS) and glycosylated hemoglobin (HbA1C) levels were also collected if possible.

LDL-C (hereafter referred to as LDL-C^DM^) was directly and quantitatively measured using the SIEMENS Dimension Vista^®^ System. It is a homogenous method with a two-reagent format; detergent 1 solubilizes non-LDL particles only. The yielded cholesterol is then hydrolyzed by cholesterol esterase (CE) and oxidized by cholesterol oxidase (CO) in a non-color forming reaction. Detergent 2 then solubilizes the remaining LDL particles. LDL-C, in its soluble form, undergoes hydrolysis and oxidation by CE and CO, respectively, resulting in cholestenone and hydrogen peroxide (H2O2). Catalyzed by peroxidase, H2O2 gives color in the presence of *N,N*-bis (4-sulfobutyl)-m-toluidine, disodium salt (DSBmT), and 4-aminoantipyrine (4-AA) which is measured by a bichromatic (540, 700 nm) endpoint technique. The formed color is directly proportional to the amount of LDL-C within the sample. All other parameters were directly quantified in the central hospital laboratory using the same SIEMENS autoanalyzer Dimension Vista^®^ System.

### Statistical analysis

Normality of the tested variables was analyzed by Kolmogorov–Smirnov test. The variables deviated from the expected normality, hence were explained as median with interquartile range (IQR). The 2 cohorts were compared by Mann–Whitney and chi-square (χ^2^) tests. The American College of Cardiology/American Heart Association (ACC/AHA) guideline cutoffs were used to classify samples as those with elevated LDL-C, hypertriglyceridemia, and mixed hyperlipidemia, and for obtaining TG, non-HDL-C, and LDL-C categories used for the analysis^60–62^. We first looked into the distribution of TG:VLDL-C ratio across different TG and non-HDL-C categories as follows: TG levels were grouped into: < 1.69, 1.69–2.24, 2.25–5.63, and ≥ 5.64 mmol/L, and non-HDL-C levels into: ≤ 2.57, 2.58–3.22, 3.23–3.86, 3.87–4.51, 4.52–5.16, ≥ 5.17 mmol/L. Multiple regression analysis was done to derive a novel equation for LDL-C estimation; hereafter referred to as equation^D^, and equation^D^-derived LDL-C values are referred to as LDL-C^D^. Paired t-test compared LDL-C^DM^ to those estimated by the equations.

Bland–Altman analysis was performed to detect the degree of agreement between the direct and calculated LDL-C levels (bias and limits of agreements (± 2SD) were calculated). The accuracy of the equations was determined by their ability to correctly classify LDL-C according to guideline-stratified categories of: ≤ 1.92, 1.93–2.57, 2.58–3.22, 3.23–3.86, 3.87–4.5, ≥ 4.5 mmol/L^59^. The differences between LDL-C values by the two methods was estimated as: Δ LDL-C^calculated^–LDL-C^DM^ (mmol/L), and the proportion of subjects with magnitude of errors of < 0.13, 0.13–0.24, 0.25–0.50, 0.51–0.76, and ≥ 0.77 mmol/L stratified by the aforementioned LDL-C categories were calculated^54^. A similar sub-analysis was performed on a group of LDL-C ≥ 4.9 mmol/L. To further validate the performance of the equations across different TG levels, the absolute errors were also calculated in the TG-stratified groups. The correlation between direct and estimated LDL-C in these groups were assessed by a linear regression. Data analysis was done using IBM SPSS Statistics software version 28.0.0 (SPSS Inc., Chicago, IL, USA), and statistical significance was set at a P-value of < 0.05 for all tests.

### Supplementary Information

Supplementary Information 1.

Supplementary Information 2.

## Data Availability

The dataset analysed during the current study is available from the corresponding author on reasonable request.
