# Supplementary material for: A novel equation for the estimation of low-density lipoprotein cholesterol in the Saudi Arabian population: a derivation and validation study
Source: Sci Rep. 2024 Mar 5;14:5478. doi: 10.1038/s41598-024-55921-w (PMC10914719; doi:10.1038/s41598-024-55921-w)
Supplement: Supplementary file 2 — Supplementary Information 2. [file 41598_2024_55921_MOESM2_ESM.pdf]

Supplementary File for

A Novel Equation for the Estimation of Low-Density Lipoprotein Cholesterol in the Saudi Arabian Population: A Derivation and Validation Study

Dena A. Nuwaylati[1,*], Zuhier A. Awan[1,2]

[1]Clinical Biochemistry Department, Faculty of Medicine, University of Jeddah, Jeddah 21959, Saudi Arabia

[2]Clinical Biochemistry Department, Faculty of Medicine, King Abdulaziz University, Jeddah 21465, Saudi Arabia

*Corresponding author: Dena A. Nuwaylati, Department of Clinical Biochemistry, University of Jeddah, Jeddah 21959, Saudi Arabia;

Email: dnuwaylati@uj.edu.sa; Tel: 00966-566612092

Content:

- **Table S1.** Classification of LDL-C, non-HDL-C, and TG categories according to ACC/AHA guidelines.
- **Table S2.** The percentage of samples by the magnitude of error between direct and calculated LDL-C for LDL-C ≥4.9 mmol/L in both cohorts
- **Table S3.** LDL-C levels estimated by the equations in TG-stratified groups and their mean bias of the whole study population.
- **Table S4**. LDL-C levels estimated by the equations in TG-stratified groups and their mean bias in both cohorts.
- **Figure S1.** The distribution of log-transformed TG:VLDL-C ratio in relation to log-transformed triglycerides by non-HDL-C categories.
- **Figure S2.** Bland-Altman plots of agreement between the directly measured and calculated LDL-C by the equations in the whole study population.
- **Figure S3.** The accuracy of the equations in classifying LDL-C according to guideline categories in the whole study population.
- **Figure S4.** The accuracy of the equations in classifying LDL-C ≥4.9 mmol/L.
- **Figures S5-S10.** Regression lines of correlation between direct and calculated LDL-C in TG-stratified groups in the whole study population and in cohort 1.

Table S1: Classification of LDL-C, non-HDL-C, and TG categories according to ACC/AHA guidelines

Category	Lipid level (mg/dl)	Lipid level (mmol/L)
LDL-C (1)		
Very low	50 – 74	1.29 – 1.92
Low	75 – 99	1.93 – 2.57
Borderline low	100 – 124	2.58 – 3.22
Borderline high	125 – 149	3.23 – 3.86
High	150 – 174	3.87 – 4.51
Very high	≥ 175	≥ 4.52
Non-HDL-C (1)		
Very low	75 – 99	1.93 – 2.57
Low	100 – 124	2.58 – 3.22
Borderline low	125 – 149	3.23 – 3.86
Borderline high	150 – 174	3.87 – 4.51
High	175 – 199	4.52 – 5.16
Very high	≥ 200	≥ 5.17
TG (2,3)		
Optimal	< 100	< 1.12
Normal	< 150	< 1.69
Borderline	150 - 199	1.69 – 2.24
High (moderate hyper TG)	200 – 499	2.25 – 5.63
Very high (severe hyper TG)	≥ 500	≥ 5.64

Abbreviations: ACC/AHA, American College of Cardiology/American Heart Association; LDL-C, low-density lipoprotein cholesterol; Non-HDL-C, non-high-density lipoprotein cholesterol; TG, triglycerides.
Note: due to the overlapping TG levels in the first 2 categories (optimal and normal), which are both normal, we have considered all levels <1.69 mmol/L as normal and was reported as a 1 group when TG-stratified groups were analyzed in this study.

Table S2. The percentage of samples by the magnitude of error between direct and calculated LDL-C for LDL-C >4.9 mmol/L in both cohorts

	Percentage of samples in the whole population				Percentage of samples in cohort 1				Percentage of samples in cohort 2			
**Absolute error (mmol/L)**	**Equation[D]**	**Friedewald**	**Martin-Hopkins**	**Sampson-NIH**	**Equation[D]**	**Friedewald**	**Martin-Hopkins**	**Sampson-NIH**	**Equation[D]**	**Friedewald**	**Martin-Hopkins**	**Sampson-NIH**
**LDL-C >4.9 mmol/L**												
	**n = 120**				**n = 81**				**n = 39**			
**< 0.13**	26.7	19.2	23.3	20.8	21.0	13.6	17.3	20.8	38.5	30.8	35.9	30.8
**0.13 – 0.24**	21.7	17.5	16.7	20.0	21.0	18.5	18.5	20.0	23.1	15.4	12.8	23.1
**0.25 – 0.50**	34.2	34.2	35.8	32.5	37.0	37.0	37.0	32.5	28.2	28.2	33.3	28.2
**0.51 – 0.76**	12.5	13.3	15.0	13.3	14.8	11.1	14.8	13.3	7.7	17.9	15.4	12.8
**> 0.77**	5.0	15.8	9.2	13.3	6.2	19.8	12.3	13.3	2.6	7.7	2.6	5.1

The numbers present the percentage of subjects in each category.

Table S3. LDL-C levels estimated by the equations in TG-stratified groups and their mean bias in the whole study population

Estimating method	LDL-C (mmol/L)	Mean bias (mmol/L)	*P* value
**TG < 1.69 mmol/l (n = 1290)**			
Direct measurement	2.86 ± 0.94 (2.81 – 2.92)	-	
Equation^{D}	2.86 ± 0.89 (2.81 – 2.91)	-0.00 ± 0.20 (-0.01 – 0.01)	0.610
Equation^{F}	2.72 ± 0.96 (2.67 – 2.77)	-0.14 ± 0.22 (-0.15 – -0.13)	< 0.001
Equation^{M}	2.70 ± 0.94 (2.65 – 2.75)	-0.16 ± 0.21 (-0.17 – -0.15)	< 0.001
Equation^{S}	2.76 ± 0.98 (2.7 – 2.81)	-0.11 ± 0.22 (-0.12 – -0.10)	< 0.001
**TG 1.69 – 2.24 mmol/l (n = 426)**			
Direct measurement	3.37 ± 1.13 (3.26 – 3.48)	-	-
Equation^{D}	3.36 ± 1.27 (3.23 – 3.48)	-0.01 ± 0.43 (-0.05 – 0.03)	0.559
Equation^{F}	3.09 ± 1.38 (2.96 – 3.22)	-0.28 ± 0.50 (-0.33 – -0.23)	< 0.001
Equation^{M}	3.22 ± 1.33 (3.09 – 3.34)	-0.15 ± 0.48 (-0.20 – -0.11)	< 0.001
Equation^{S}	3.19 ± 1.35 (3.06 – 3.32)	-0.19 ± 0.47 (-0.23 – -0.14)	< 0.001
**TG 2.25 – 5.63 mmol/l (n = 502)**			
Direct measurement	3.47 ± 1.18 (3.37 – 3.57)	-	-
Equation^{D}	3.44 ± 1.18 (3.33 – 3.54)	-0.03 ± 0.38 (-0.06 – 0.00)	0.084
Equation^{F}	2.95 ± 1.28 (2.84 – 3.06)	-0.51 ± 0.42 (-0.55 – -0.48)	< 0.001
Equation^{M}	3.30 ± 1.17 (3.20 – 3.40)	-0.16 ± 0.38 (-0.20 – -0.13)	< 0.001
Equation^{S}	3.12 ± 1.19 (3.01 – 3.22)	-0.35 ± 0.38 (-0.39 – -0.32)	< 0.001
**TG ≥ 5.64 mmol/l (n = 27)**			
Direct measurement	3.39 ± 1.08 (2.96 – 3.82)	-	-
Equation^{D}	3.47 ± 1.05 (3.05 – 3.89)	0.09 ± 0.60 (-0.15 – 0.32)	0.456
Equation^{F}	2.28 ± 1.06 (1.86 – 2.70)	-1.11 ± 0.55 (-1.33 – -0.89)	< 0.001
Equation^{M}	3.29 ± 0.93 (3.66 – 2.92)	-0.10 ± 0.55 (-0.31 – 0.12)	0.367
Equation^{S}	2.67 ± 0.81 (2.35 – 2.99)	-0.72 ± 0.55 (-0.93 – -0.50)	< 0.001

Data presented as mean ± SD (95%CI). *P* values are of paired t-test between each direct and calculated LDL-C values. LDL-C, low-density lipoprotein cholesterol; Equation^{D}, novel equation; Equation^{F}, Friedewald equation; Equation^{M}, Martin-Hopkins equation; Equation^{S}, Sampson-NIH equation.

Table S4. LDL-C levels estimated by the equations in TG-stratified groups and their mean bias in both cohorts

Estimating method	LDL-C (mmol/L)	Mean bias (mmol/L)	*P* value
Cohort 1			
**TG < 1.69 mmol/l (n = 846)**			
Direct measurement	2.85 ± 0.92 (2.79 – 2.91)		
Equation^{D}	2.84 ± 0.879 (2.78 – 2.90)	-0.01 ± 0.20 (-0.02 – 0.01)	0.251
Equation^{F}	2.71 ± 0.94 (2.64 – 2.77)	-0.15 ± 0.22 (-0.16 – -0.13)	< 0.001
Equation^{M}	2.69 ± 0.92 (2.63 – 2.75)	-0.16 ± 0.21 (-0.18 – -0.15)	< 0.001
Equation^{S}	2.74 ± 0.96 (2.68 – 2.81)	-0.11 ± 0.22 (-0.13 – -0.10)	< 0.001
**TG 1.69 – 2.24 mmol/l (n = 277)**			
Direct measurement	3.31 ± 1.17 (3.17 – 3.45)	-	
Equation^{D}	3.31 ± 1.37 (3.14 – 3.47)	-0.00 ± 0.49 (-0.06 – 0.05)	0.900
Equation^{F}	3.04 ± 1.49 (2.86 – 3.21)	-0.28 ± 0.57 (-0.34 – -0.21)	< 0.001
Equation^{M}	3.16 ± 1.43 (2.99 – 3.33)	-0.15 ± 0.55 (-0.21 – -0.08)	< 0.001
Equation^{S}	3.14 ± 1.46 (2.96 – 3.31)	-0.18 ± 0.55 (-0.24 – -0.11)	< 0.001
**TG 2.25 – 5.63 mmol/l (n = 356)**			
Direct measurement	3.52 ± 1.14 (3.40 – 3.63)	-	
Equation^{D}	3.47 ± 1.13 (3.35 – 3.59)	-0.04 ± 0.37 (-0.08 – -0.00)	0.028
Equation^{F}	2.99 ± 1.23 (2.86 – 3.12)	-0.53 ± 0.40 (-0.57 – -0.49)	< 0.001
Equation^{M}	3.33 ± 1.12 (3.22 – 3.45)	-0.18 ± 0.37 (-0.22 – -0.14)	< 0.001
Equation^{S}	3.16 ± 1.14 (3.03 – 3.27)	-0.37 ± 0.37 (-0.41 – -0.33)	< 0.001
**TG ≥ 5.64 mmol/l (n = 18)**			
Direct measurement	3.34 ± 1.22 (2.73 – 3.95)	-	
Equation^{D}	3.50 ± 1.20 (2.89 – 4.10)	0.16 ± 0.62 (-0.15 – 0.46)	0.292
Equation^{F}	2.31 ± 1.19 (1.71 – 2.90)	-1.03 ± 0.56 (-1.3 – -0.75)	< 0.001
Equation^{M}	3.32 ± 1.06 (2.79 – 3.85)	-0.01 ± 0.57 (-0.30 – 0.27)	0.915
Equation^{S}	2.69 ± 0.91 (2.24 – 3.14)	-0.66 ± 0.58 (-0.95 – -0.36)	< 0.001
Cohort 2			
**TG < 1.69 mmol/l (n = 444)**			
Direct measurement	2.87 ± 0.96 (2.78 – 2.96)	-	
Equation^{D}	2.88 ± 0.93 (2.79 – 2.97)	0.01 ± 0.20 (-0.01 – 0.03)	0.472
Equation^{F}	2.74 ± 1 (2.65 – 2.84)	-0.13 ± 0.22 (-0.15 – -0.11)	< 0.001
Equation^{M}	2.73 ± 0.98 (2.63 – 2.82)	-0.15 ± 0.21 (-0.17 – -0.13)	< 0.001
Equation^{S}	2.78 ± 1.02 (2.69 – 2.88)	-0.10 ± 0.22 (-0.12 – -0.08)	< 0.001
**TG 1.69 – 2.24 mmol/l (n = 149)**			
Direct measurement	3.47 ± 1.03 (3.31 – 3.64)	-	
Equation^{D}	3.44 ± 1.06 (3.27 – 3.62)	-0.03 ± 0.28 (-0.07 – 0.02)	0.222
Equation^{F}	3.18 ± 1.15 (3 – 3.37)	-0.29 ± 0.31 (-0.34 – -0.24)	< 0.001
Equation^{M}	3.3 ± 1.09 (3.12 – 3.482)	-0.17 ± 0.29 (-0.22 – -0.13)	< 0.001
Equation^{S}	3.28 ± 1.12 (3.10 – 3.46)	-0.20 ± 0.30 (-0.24 – -0.15)	< 0.001

**TG 2.25 – 5.63 mmol/l (n = 146)**			
Direct measurement	3.34 ± 1.24 (3.14 – 3.54)	-	
Equation[D]	3.34 ± 1.27 (3.14 – 3.55)	0.01 ± 0.39 (-0.06 – 0.07)	0.875
Equation[F]	2.86 ± 1.39 (2.63 – 3.08)	-0.48 ± 0.45 (-0.56 – -0.41)	< 0.001
Equation[M]	3.22 ± 1.28 (3.013 – 3.43)	-0.12 ± 0.40 (-0.19 – -0.06)	< 0.001
Equation[S]	3.03 ± 1.29 (2.82 – 3.24)	-0.31 ± 0.41 (-0.38 – -0.25)	< 0.001
**TG ≥ 5.64 mmol/l (n = 9)**			
Direct measurement	3.48 ± 0.77 (2.88 – 4.08)	-	
Equation[D]	3.42 ± 0.71 (2.88 – 3.97)	-0.06 ± 0.54 (-0.47 – 0.36)	0.756
Equation[F]	2.22 ± 0.79 (1.61 – 2.83)	-1.27 ± 0.53 (-1.67 – -0.85)	< 0.001
Equation[M]	3.22 ± 0.62 (2.74 – 3.70)	-0.26 ± 0.50 (-0.64 – 0.12)	0.155
Equation[S]	2.65 ± 0.61 (2.18 – 3.12)	-0.84 ± 0. 50 (-1.22 – -0.46)	< 0.001

Data presented as mean ± SD (95%CI). *P* values are of paired t-test between each direct and calculated LDL-C values. LDL-C, low-density lipoprotein cholesterol; Equation[D], novel equation; Equation[F], Friedewald equation; Equation[M], Martin-Hopkins equation; Equation[S], Sampson-NIH equation.

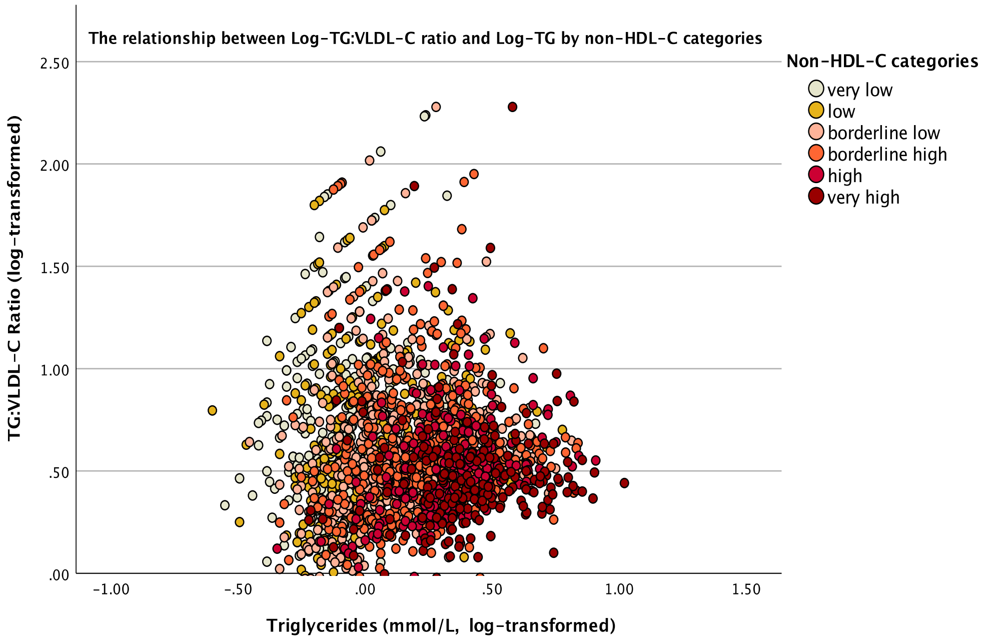

Figure S1. The distribution of log-transformed TG:VLDL-C ratio in relation to log-transformed triglycerides by non-HDL-C categories.

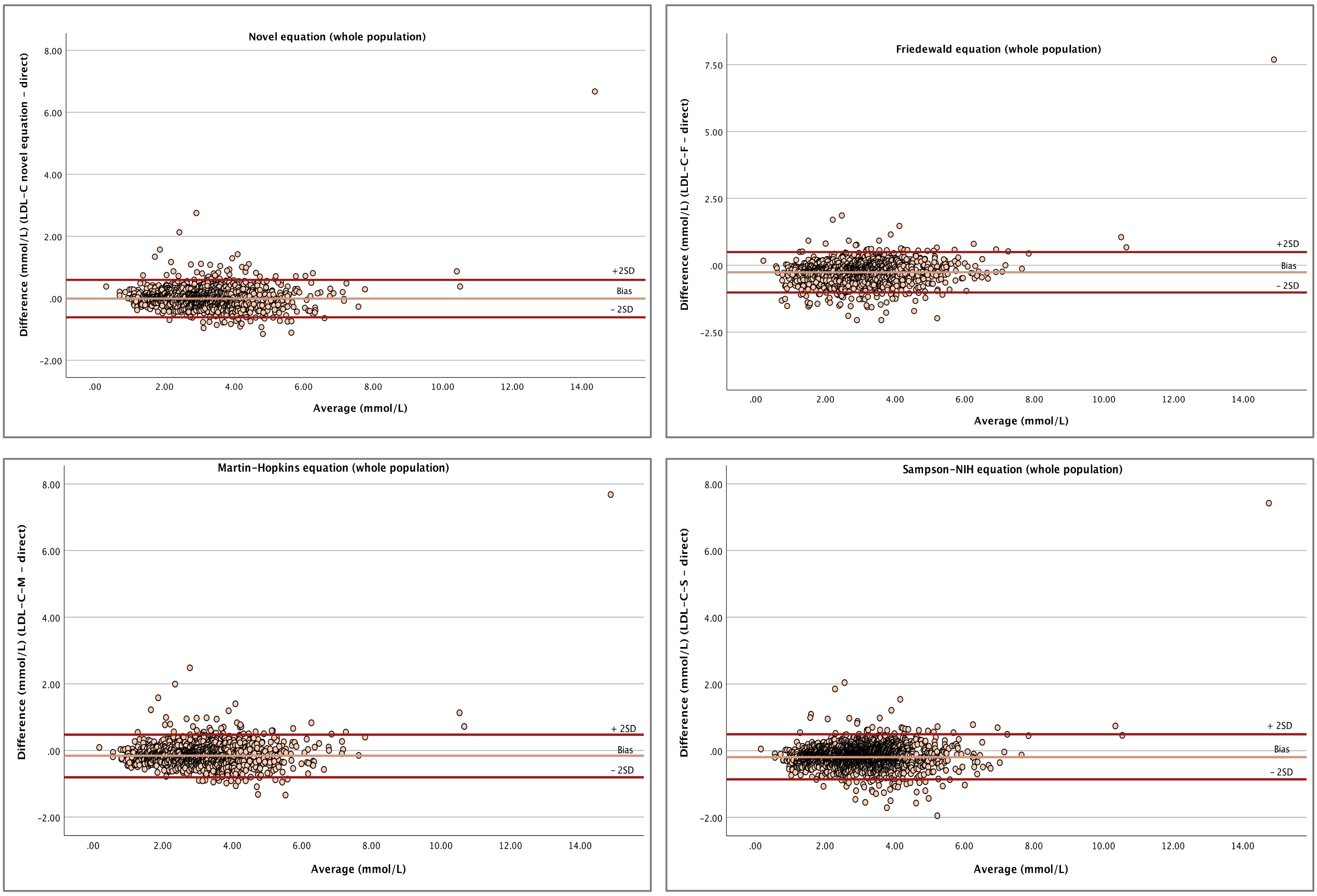

Figure S2. Bland-Altman plots of agreement between the directly measured and calculated LDL-C by the equations in the whole study population. The lines demonstrate the mean bias and 95% limits of agreement (± 2SD). LDL-C; low-density lipoprotein cholesterol; SD, standard deviation.

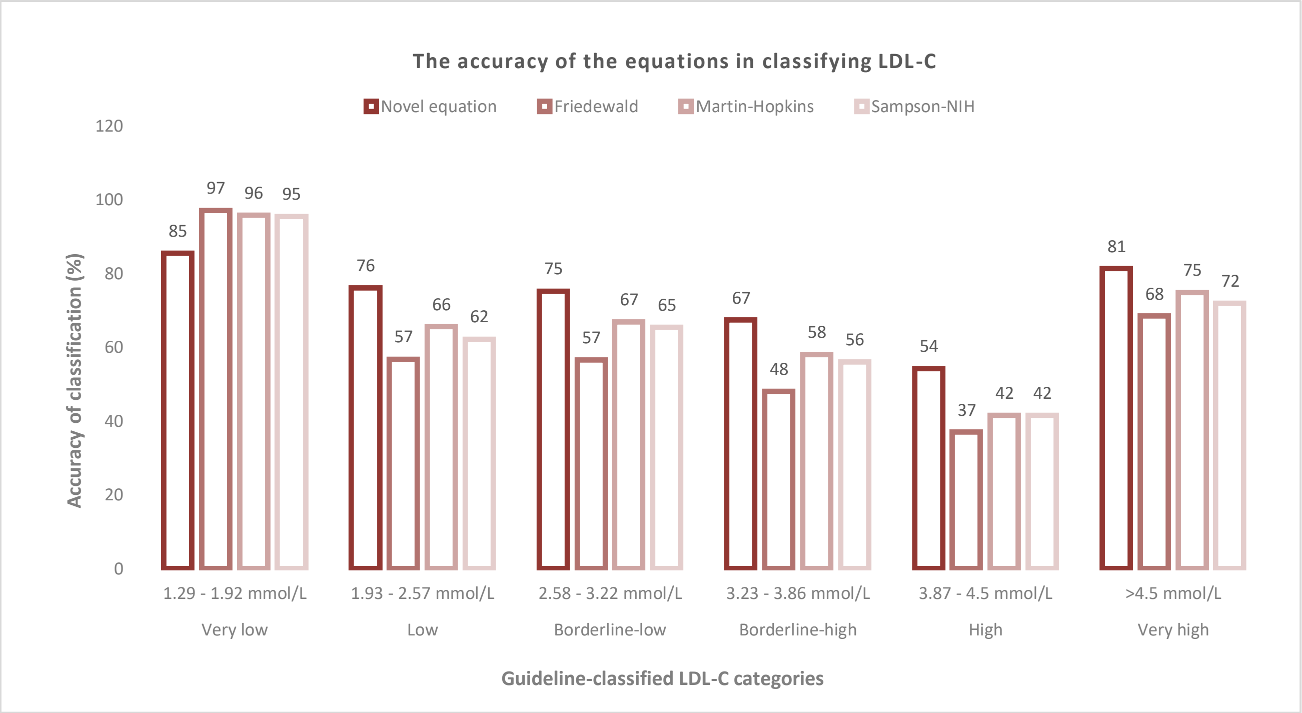

Figure S3. The accuracy of the equations in classifying LDL-C according to guideline categories in the whole study population.

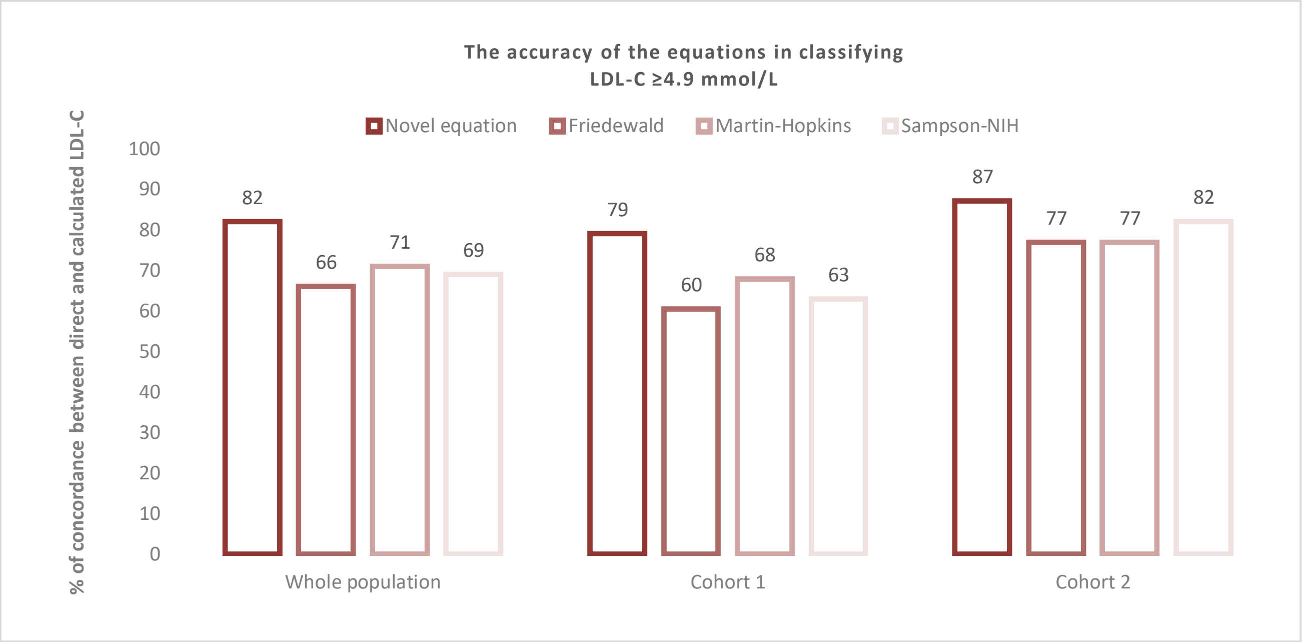

Figure S4. The accuracy of the equations in classifying LDL-C $\geq$4.9 mmol/L.

TG <1.69 mmol/L

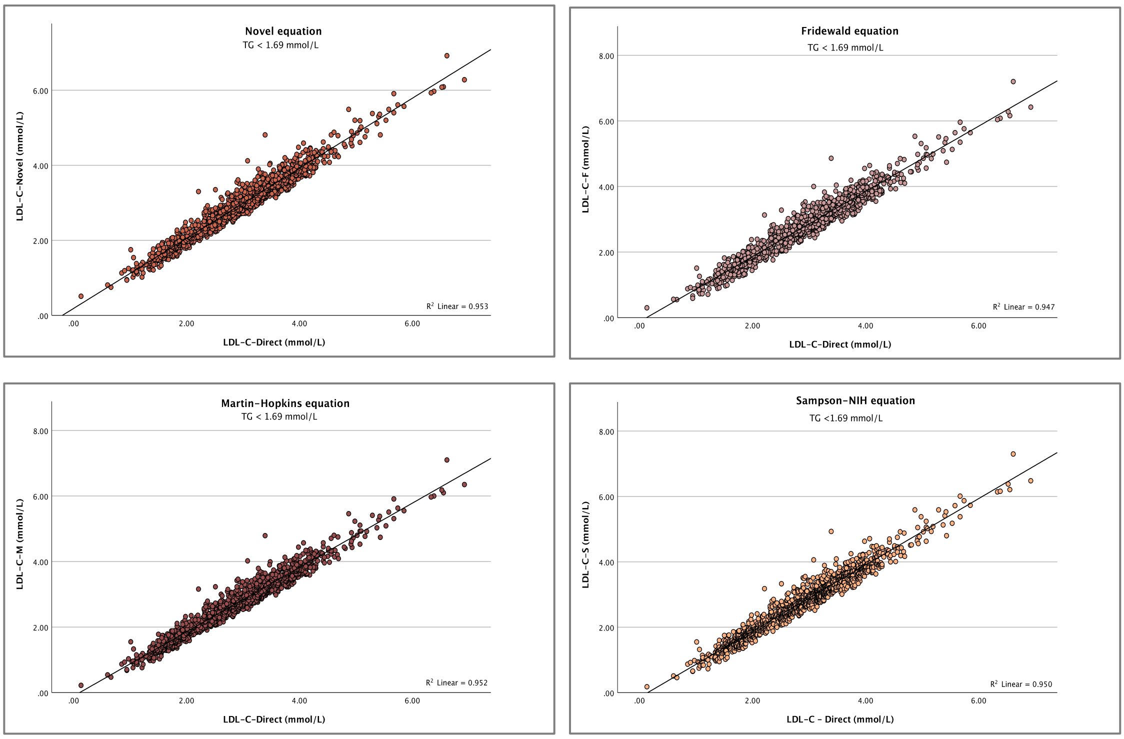

Figure S5. Regression lines of correlation between direct and calculated LDL-C in TG <1.69 mmol/L in the whole study population.

TG 1.69 – 2.24 mmol/L

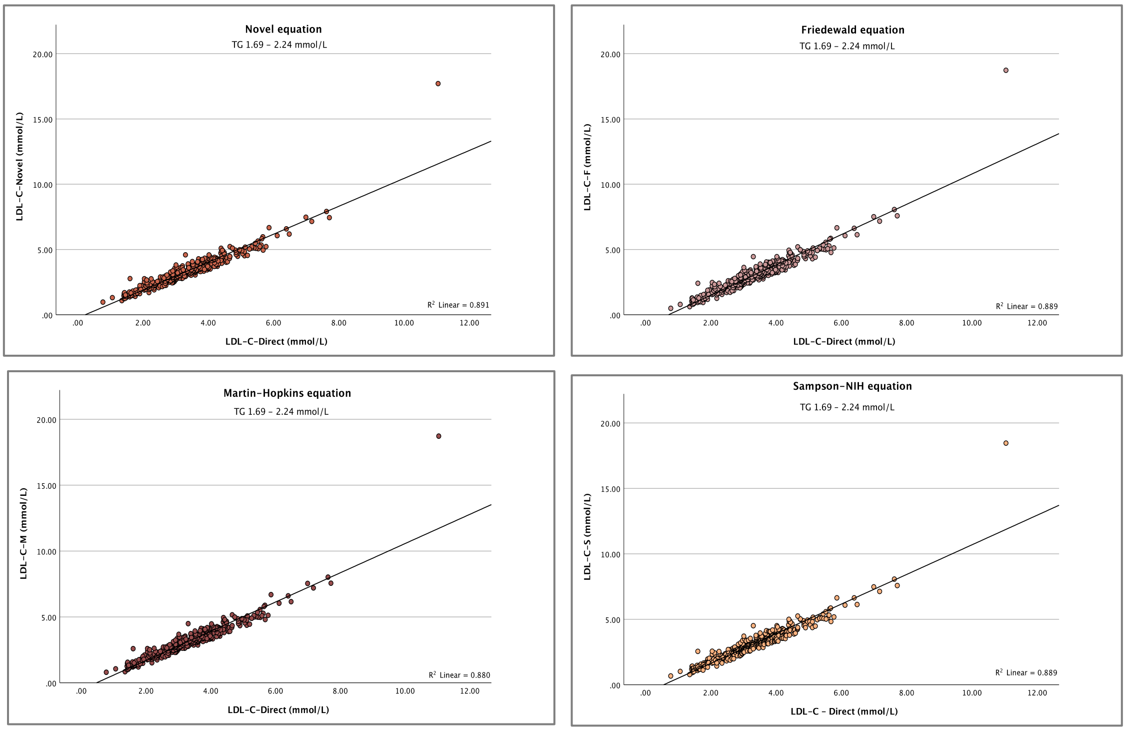

Figure S6. Regression lines of correlation between direct and calculated LDL-C in TG 1.69 – 2.24 mmol/L in the whole study population.

TG 2.25 – 5.63 mmol/L

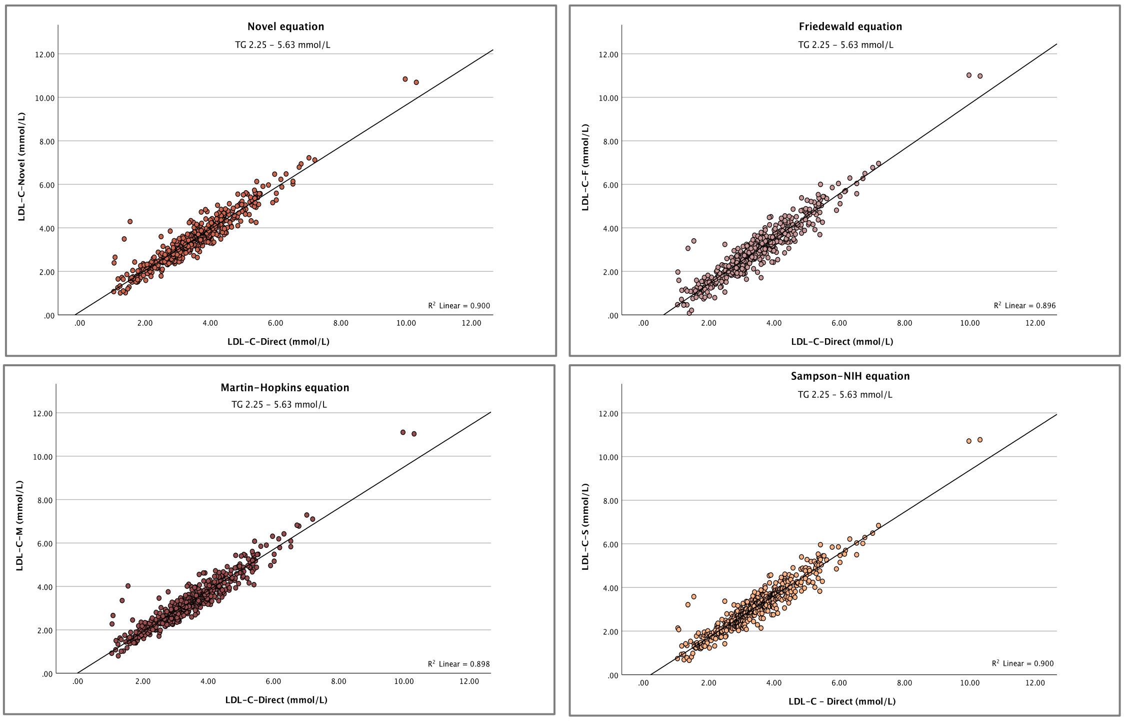

Figure S7. Regression lines of correlation between direct and calculated LDL-C in TG 2.25 – 5.63 mmol/L in the whole study population.

TG <1.69 mmol/L

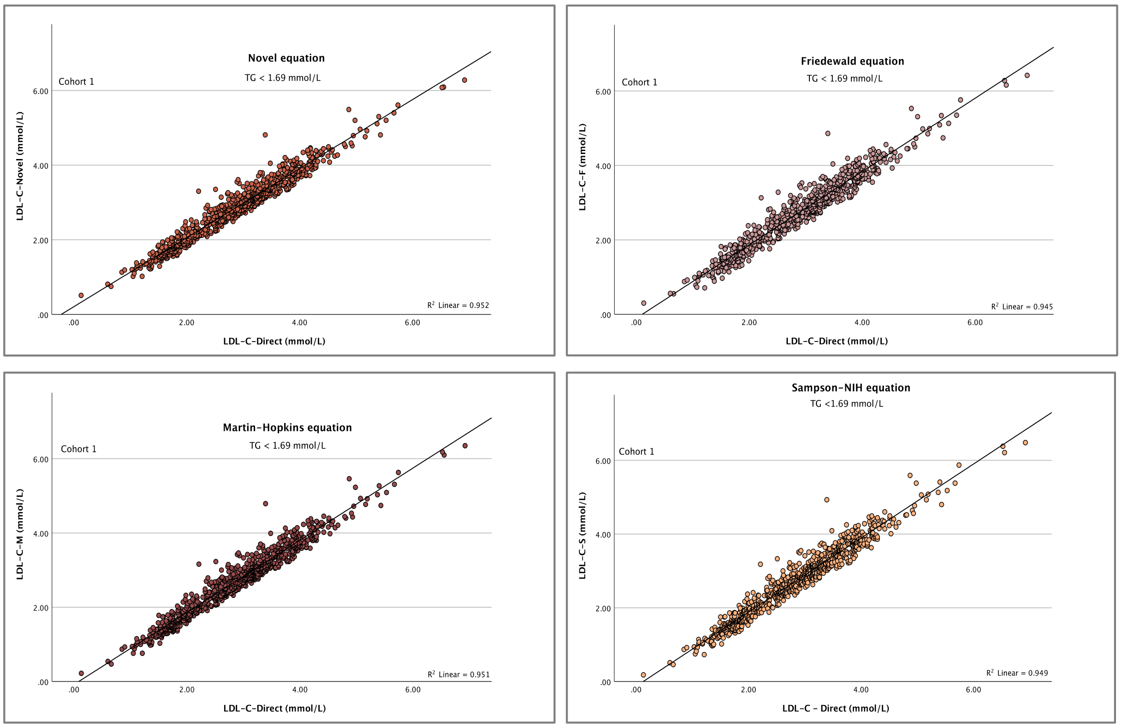

Figure S8. Regression lines of correlation between direct and calculated LDL-C in TG <1.69 mmol/L in cohort 1.

TG 1.69 – 2.24 mmol/L

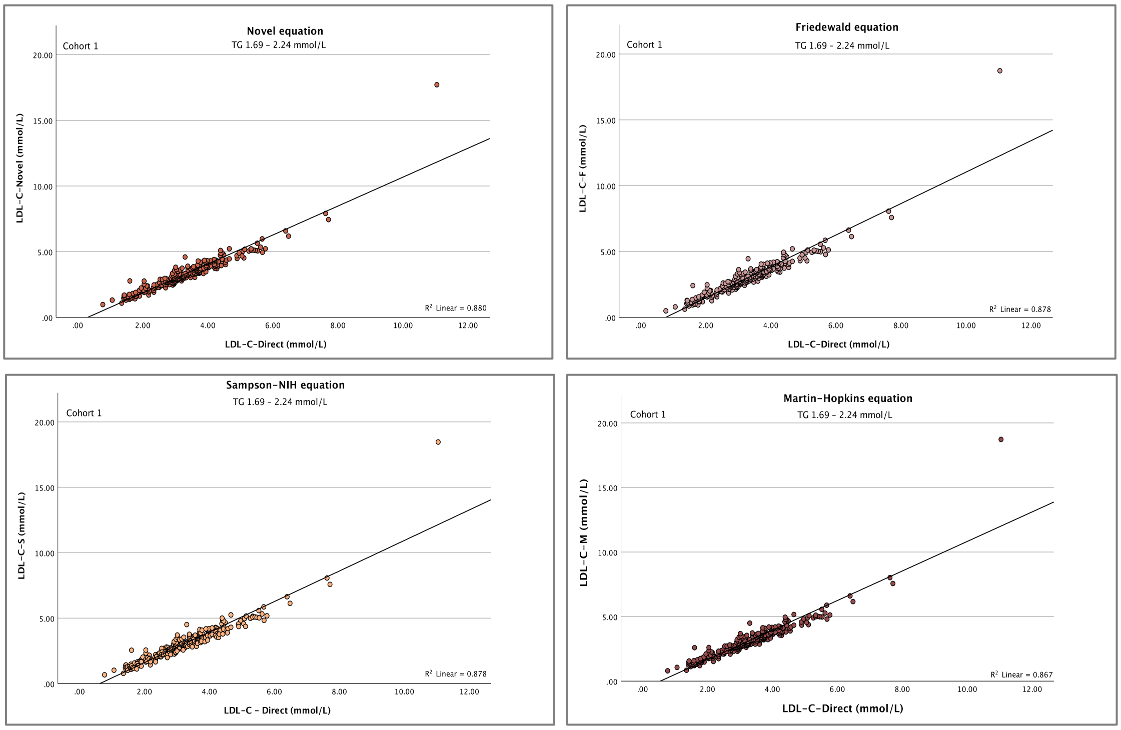

Figure S9. Regression lines of correlation between direct and calculated LDL-C in TG 1.69 – 2.24 mmol/L in cohort 1.

TG 1.69 – 2.24 mmol/L

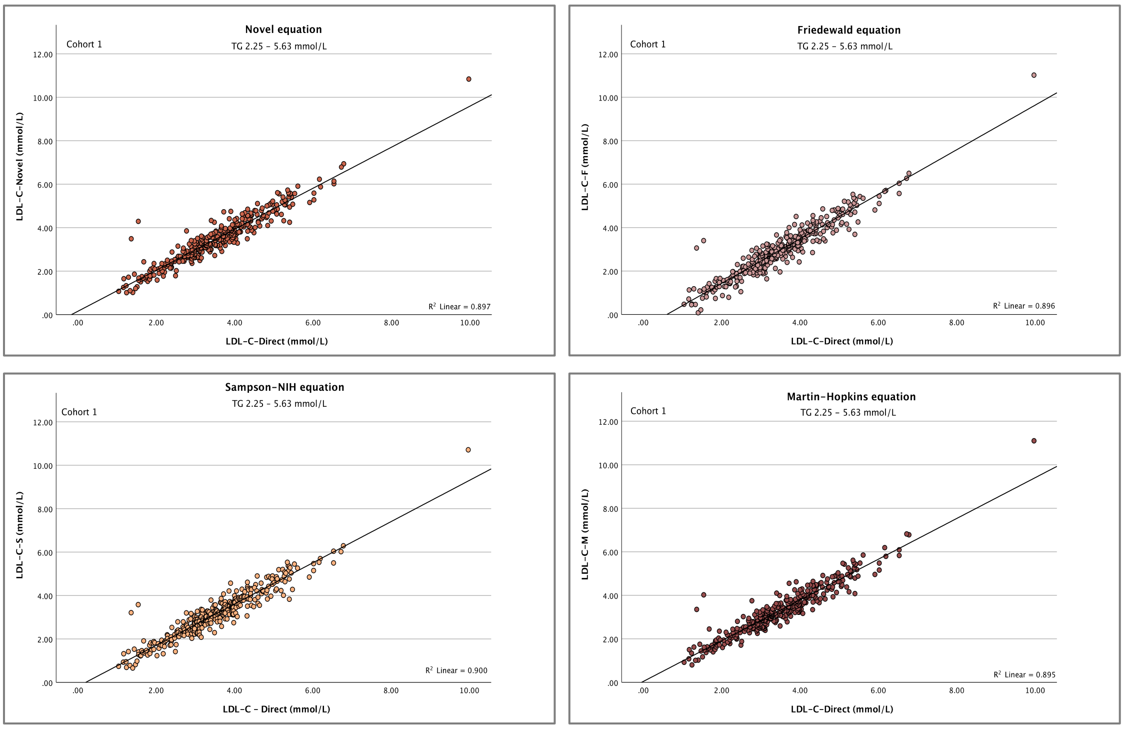

Figure S10. Regression lines of correlation between direct and calculated LDL-C in TG 2.25 – 5.63 mmol/L in cohort 1.

References

1. Smith, S. C., Jr. & Grundy, S. M. 2013 ACC/AHA guideline recommends fixed-dose strategies instead of targeted goals to lower blood cholesterol. *J Am Coll Cardiol* **64,** 601-612 (2014).
2. Miller, M. *et al.* Triglycerides and cardiovascular disease: a scientific statement from the American Heart Association. *Circulation* **123,** 2292-2333 (2011).
3. Skulas-Ray, A. C. *et al.* Omega-3 fatty acids for the management of hypertriglyceridemia: a science advisory from the American Heart Association. *Circulation* **140,** e673-e691 (2019).
